# Association of Coexisting Hypertension and Diabetes with Blood Gas Analysis Indicators in COPD Patients: A Propensity Score-Matched Analysis

**DOI:** 10.3390/healthcare14162500

**Published:** 2026-08-12

**Authors:** Jiayi Cui, Zhizhou Duan, Jun Wang

**Affiliations:** 1 Jiangxi Medical College, Nanchang University, Nanchang 330031, China; cuijiayi1002@163.com; 2 The Second Department of Respiratory Disease, Jiangxi Provincial People’s Hospital, The First Affiliated Hospital of Nanchang Medical College, Nanchang 330006, China; 3 Preventive Health Service, Jiangxi Provincial People’s Hospital, The First Affiliated Hospital of Nanchang Medical College, Nanchang 330006, China; 4 JXHC Key Laboratory of Respiratory Diseases, Nanchang 330006, China

**Keywords:** propensity score matching (PSM), observational studies, COPD patients, comorbid hypertension and diabetes

## Abstract

**Background**: Hypertension and diabetes commonly coexist in patients with chronic obstructive pulmonary disease (COPD), but their associations with arterial blood gas parameters remain insufficiently characterized, particularly after accounting for measured differences in baseline characteristics. This study aimed to examine the associations between coexisting hypertension and diabetes and selected arterial blood gas parameters in older patients with COPD. Propensity score matching (PSM) was used to improve the comparability of the study groups and reduce confounding from measured baseline covariates. **Patients and Methods**: A retrospective cross-sectional study was conducted involving 1030 patients. The comorbidity group comprised COPD patients with both hypertension and diabetes (*n* = 340), while the COPD-only group refers to COPD patients without hypertension or diabetes (*n* = 690). Propensity score matching (PSM) was estimated using a regression model with covariates including age, sex, marital status, BMI, temperature, and pulse. Balance was assessed using standardized mean difference (SMD), and linear regression models were adopted to evaluate the association between coexisting hypertension and diabetes and each blood gas indicator (PCO_2_, PO_2_, SaO_2_). **Results**: We performed 1:1 nearest-neighbor matching and successfully generated 340 matched pairs, forming a total matched cohort of 680 patients; all unmatched participants from the original 1030 subjects were excluded from subsequent outcome comparisons. Covariate balance was optimized for most baseline indicators after PSM, although mild residual imbalance remained in age and BMI after matching. The comorbidity group demonstrated a statistically significant reduction in PCO_2_ (B: −2.54, 95% CI: −4.52 to −0.56) compared to the COPD-only group, while no significant differences were observed in PO_2_ (B: −2.48, 95% CI: −7.6 to 2.64) or SaO_2_ (B: −1.16, 95% CI: −2.67 to 0.35). In addition, a sensitivity analysis adjusting for age and BMI was conducted to evaluate the robustness of the findings. **Conclusions**: After propensity score matching, coexisting hypertension and diabetes were associated with lower PCO_2_ levels in elderly COPD patients, while no significant associations were found for PO_2_ or SaO_2_.

## 1. Introduction

The coexistence of hypertension and diabetes represents a significant and growing challenge in global public health, particularly among the aging population [1,2]. These conditions frequently cluster, with shared pathophysiological pathways such as chronic inflammation, insulin resistance, and endothelial dysfunction leading to cumulative damage across multiple organ systems, including the heart, kidneys, and lung [3]. Chronic obstructive pulmonary disease (COPD) is a heterogeneous respiratory disorder characterized by persistent airflow limitation, recurrent exacerbations, and a substantial burden of systemic comorbidity [4]. Hypertension and diabetes are among the most frequent cardiometabolic conditions observed in patients with COPD [5,6,7].

Previous research demonstrated that patients with GOLD stage 3–4 COPD exhibited elevated prevalence of diabetes (OR = 1.5, 95% CI: 1.1–1.9) and hypertension (OR = 1.6, 95% CI: 1.3–1.9) [5]. This triple comorbidity generates a complex clinical phenotype that may exacerbate adverse impacts on overall physical condition and disease progression. The synergistic correlation among these three conditions substantially elevates the risk of end-organ injury involving the heart, brain and kidneys, rendering this multimorbidity a critical public health priority [8].

Respiratory function, as objectively assessed by arterial blood gas analysis, serves as a critical indicator of overall physiological reserve and disease severity in elderly patients with multiple comorbidities. Parameters such as carbon dioxide partial pressure (PCO_2_), oxygen partial pressure (PO_2_), and oxygen saturation (SaO_2_) provide invaluable insights into ventilatory efficiency, gas exchange capability, and acid–base balance [9]. In patients with multimorbidity, changes in these blood gas parameters may not merely reflect COPD progression; instead, they can be substantially shaped by the presence and severity of concomitant cardiovascular and metabolic disorders [10,11]. A study revealed that patients with both COPD and T2DM exhibited higher arterial PCO_2_ levels compared to those with COPD alone. Furthermore, T2DM risk increased with rising PCO_2_ levels [12]. For instance, type 2 diabetes is associated with impaired pulmonary function, further affecting PCO_2_ or PO_2_ levels [13]. Likewise, metabolic load and microvascular complications associated with diabetes and hypertension may correlate with altered oxygen transport and peripheral tissue utilization, which in turn is linked to shifts in arterial PO_2_ and SaO_2_ levels.

Observational research better mirrors routine real-world clinical practice and boasts practical strengths, including reduced research expenditure and minimal ethical constraints, making such designs broadly feasible for clinical investigations. Nevertheless, confounding bias poses a persistent threat to internal validity in non-randomized analyses [14]. Confounding arises from baseline covariates that correlate simultaneously with the exposure of interest and the measured study outcomes. Without random group allocation, systematic disparities in demographic and clinical features inevitably emerge between patients with versus without the target comorbidity. Such baseline gaps imply that any detected statistical relationship between exposure and blood gas endpoints may stem entirely from pre-existing intergroup differences, rather than representing an authentic biological link. Existing observational datasets frequently carry pronounced baseline imbalance across key clinical variables, hindering efforts to distinguish genuine physiological correlations from spurious associations induced by confounders. Not all individuals with COPD develop concurrent hypertension and type 2 diabetes; patients living with this dual cardiometabolic multimorbidity typically exhibit distinct baseline profiles relative to those with isolated COPD, including disparities in age, sex, BMI, insulin resistance, renal function, and cumulative cardiovascular risk burden [15,16,17,18]. This inherent heterogeneity presents research challenges: when attempting to investigate the evaluations of disease-related correlates in this multimorbid cohort are severely hampered by systematic baseline disparities that induce prominent confounding. If these confounders are not adequately addressed, any observed intergroup differences may be erroneously attributed to the “comorbid hypertension and diabetes” status itself, overlooking the complex underlying patient characteristic profile.

To address this challenge, a series of statistical methods designed to mimic randomized trials have emerged. Among these, Propensity Score Matching (PSM), introduced by Rosenbaum and Rubin in 1983 [19], has become one of the most widely used techniques in medical research. In recent years, PSM has been increasingly applied across multiple disciplines [20,21,22], including cardiology, oncology, and pulmonology. Its core principle involves calculating a single, composite propensity score for each individual based on a set of observed baseline covariates (e.g., age, sex, disease severity, comorbidities). Individuals from the two groups with similar estimated propensity scores are matched to construct a new matched study sample. In this sample, the distributions of the measured covariates included in the propensity score model are theoretically expected to be more balanced between the groups, thereby creating a “pseudo-randomized” comparison setting. By reducing observed baseline differences, propensity score matching can improve group comparability and enhance the robustness of the estimated associations and study findings. Hence, when randomized controlled trials cannot be adequately conducted due to ethical constraints, poor patient compliance, or other limitations, propensity score matching (PSM) can be employed in observational studies to simulate a quasi-randomized setting, which not only facilitates the implementation of clinical research but also enhances the robustness of the conclusions [23].

Arterial blood gas parameters have been shown to reflect clinically relevant respiratory impairment in COPD. Delclaux et al. reported that blood gas abnormalities in elderly patients with COPD were associated with airflow obstruction, with PO_2_ positively correlated with FEV_1_ and PCO_2_ negatively correlated with FEV_1_ [24]. Recent evidence has also suggested that PCO_2_ may be related to the risk of developing type 2 diabetes in patients with COPD [12]. Nevertheless, whether COPD patients with coexisting hypertension and diabetes have distinct blood gas profiles, including PCO_2_, PO_2_, and SaO_2_, compared with COPD-only patients remains insufficiently characterized. In particular, few studies have examined this question after accounting for baseline differences between patients with and without these cardiometabolic comorbidities. Therefore, this observational cross-sectional study applied propensity score matching to improve baseline comparability between COPD patients with coexisting hypertension and diabetes and COPD-only patients. We aimed to examine the association between coexisting hypertension and diabetes and arterial blood gas parameters in elderly patients with COPD after adjustment for available measured covariates. The findings may provide preliminary evidence for understanding blood gas profiles in COPD patients with cardiometabolic comorbidities.

## 2. Material and Methods

### 2.1. Study Population and Data Collection

In this population-based cross-sectional study, data from COPD patients treated in the Respiratory Department of Jiangxi Provincial People’s Hospital (The First Affiliated Hospital of Nanchang Medical College) between 2019 and 2024 through the case inquiry system were analyzed. Demographic characteristics, vital signs, comorbidity status, and arterial blood gas parameters were extracted from the electronic medical record system.

From the initial database, we have identified patients with a primary diagnosis of COPD. Participants were included based on the following criteria: 1. Inclusion criteria: (1) age ≥ 60 years; (2) complete arterial blood gas indicators data (PCO_2_, SaO_2_, PO_2_). 2. Exclusion criteria: (1) patients with COPD complicated by hypertension alone or diabetes alone; (2) presence of other diseases severely affecting survival or gas exchange (e.g., advanced malignant tumors, severe heart failure NYHA class IV, end-stage renal disease, respiratory failure primarily caused by non-COPD conditions); (3) duplicate hospitalization records, in which case only the first eligible record was retained for analysis. Then, patients were divided into two groups according to comorbidity status: (1) the comorbidity group refers to “COPD patients with coexisting hypertension and diabetes”, and (2) the COPD-only group refers to “COPD-only patients” without hypertension or diabetes. Among them, COPD, hypertension, and diabetes were defined according to established clinical diagnostic criteria and guideline-based diagnoses recorded in the medical records, including the Global Initiative for Chronic Obstructive Lung Disease criteria for COPD, the International Society of Hypertension criteria for hypertension, and the American Diabetes Association diagnostic criteria for diabetes [25,26,27].

Specifically, we initially retrieved 11,052 patients diagnosed with COPD from the hospital electronic medical record system. A total of 9131 subjects were excluded owing to the absence of arterial blood gas examination on admission. After further exclusion based on predefined criteria (COPD with hypertension alone or diabetes alone (*n* = 822) and age < 60 years (*n* = 69), 1030 eligible patients were included in the analysis, including 340 COPD patients with coexisting hypertension and diabetes and 690 COPD-only patients.

### 2.2. Ethical Approval and Data Privacy

The study protocol was reviewed and approved by the Ethics Committee of Jiangxi Provincial People’s Hospital (approval No. KK202508; approval date: 19 February 2025). The requirement for written informed consent was waived by the ethics committee, given that this retrospective study only adopted de-identified clinical data without any direct patient contact or additional clinical interventions. All personal identifiers of patients were eliminated prior to data analysis. This study was carried out in strict accordance with the Declaration of Helsinki and institutional administrative regulations governing retrospective clinical studies.

### 2.3. Primary Outcome Measures

The core outcome variables of this study were the following parameters from arterial blood gas analysis: (1) Partial Pressure of Carbon Dioxide (PCO_2_, mmHg). (2) Oxygen Saturation (SaO_2_, %). (3) Partial Pressure of Oxygen (PO_2_, mmHg).

### 2.4. Research Method

We conducted propensity score matching (PSM) analysis, a valuable way to control for bias and achieve pseudo-randomization in retrospective observation studies, to control confounding variables to create a balanced and comparable group [23,28]. Regression model to calculate the propensity score for each patient, which represents the conditional probability of being in the comorbidity group given their baseline covariates. The covariates were: age, sex, marital status, BMI category, body temperature and pulse rate. After obtaining the propensity scores for all patients, we performed 1:1 nearest neighbor matching and all unmatched participants were excluded from subsequent outcome comparisons. 

To evaluate the effectiveness of PSM in balancing baseline characteristics, we compared the data before and after matching using the following criteria: (1) Standardized Mean Difference (SMD): This served as the primary metric for assessing covariate balance after matching [29]. The SMD quantifies the standardized difference between groups for both continuous and binary variables. (2) Statistical Test *p*-value: As a supplementary reference, we also reported *p*-values from between-group comparisons after matching (using *t*-tests for continuous variables or and chi-square or Fisher’s exact tests for categorical variables). In addition, we can visually observe the degree of difference in propensity scores between the two groups before and after the match via the distribution of propensity score before and after PSM analysis. The overlap of the two densities between the two groups constitutes the area of overlap, also known as the area of common support [30].

### 2.5. Statistical Analysis

We utilized Excel to establish a database, and all statistical analyses were performed using R software (version 4.4.1). The propensity score matching and balance assessment were primarily implemented using the “MatchIt” package. Continuous variables were summarized as mean ± standard deviation, and categorical variables were summarized as frequencies and percentages. Missing data for temperature (°C) and pulse (beats per minute) were handled via multiple imputation. Notably, the study inclusion criteria mandated complete core baseline covariates; only temperature and pulse contained partial missing records, and these variables were not used as eligibility screening indicators. Baseline balance before and after matching was evaluated using covariate-specific SMD. For the outcome analysis, PCO_2_, PO_2_, and SaO_2_ were treated as continuous dependent variables. Linear regression models were used to estimate the association between coexisting hypertension and diabetes and each blood gas indicator. Regression coefficients (B) and 95% confidence intervals (CIs) were reported, with B representing the mean difference in the blood gas indicator between the comorbidity group and the COPD-only group.

All statistical tests were two-sided, and a *p*-value < 0.05 was considered statistically significant.

## 3. Results

### 3.1. Characteristics of Study Population

After applying the predefined inclusion and exclusion criteria, 1030 eligible COPD patients were included in the final analysis. Their baseline socio-demographic and clinical characteristics are summarized in Table 1. The mean age of the participants was 77.54 ± 9.36 years, with the majority (58.0%) being 75 years or older. Men constituted 80.8% of the population. Most participants were married (78.1%). The mean Body Mass Index (BMI) was 21.31 ± 3.24 kg/m^2^, with the largest proportion of participants (72.1%) falling within the normal weight range (18.5–24.9 kg/m^2^), followed by underweight (16.8%) and overweight (9.6%); only 1.5% were classified as having obesity. The mean vital signs and blood gas parameters were as follows: temperature 36.60 ± 0.55 °C, pulse 87.02 ± 18.09 beats per minute, PCO_2_ 45.26 ± 14.37 mmHg, SaO_2_ 92.79 ± 9.85%, and PO_2_ 84.41 ± 32.86 mmHg.

### 3.2. PSM Analysis

Prior to implementing propensity score matching, we compared the baseline characteristics of all 1030 study participants (340 in the comorbidity group and 690 in the COPD-only group). Detailed results are presented in the first half of Table 2. The analysis revealed statistically significant differences (*p* < 0.05) in several key baseline variables between the comorbidity group and the COPD-only group before matching. The most prominent differences were observed in age (*p* < 0.001) and BMI (*p* < 0.001) category. Patients in the comorbidity group were older on average and exhibited a significantly different BMI distribution compared to the COPD-only group. Additionally, pulse rate (*p* = 0.015) also showed a significant difference between the two groups. These marked baseline imbalances confirmed that directly comparing outcomes between the two groups in subsequent analyses would introduce substantial confounding bias, thereby underscoring the necessity of using PSM. The results of the baseline characteristic comparison after matching are listed in the second half of Table 2. PSM demonstrated significant effectiveness in balancing most variables. After matching, between-group differences for variables such as marital status (*p* = 0.089), and pulse (*p* = 0.193) were no longer statistically significant (*p* > 0.05), showing that these variables achieved balance in the matched sample (SMD for each covariate before and after matching is shown in Supplementary Table S1).

**Figure 1:**
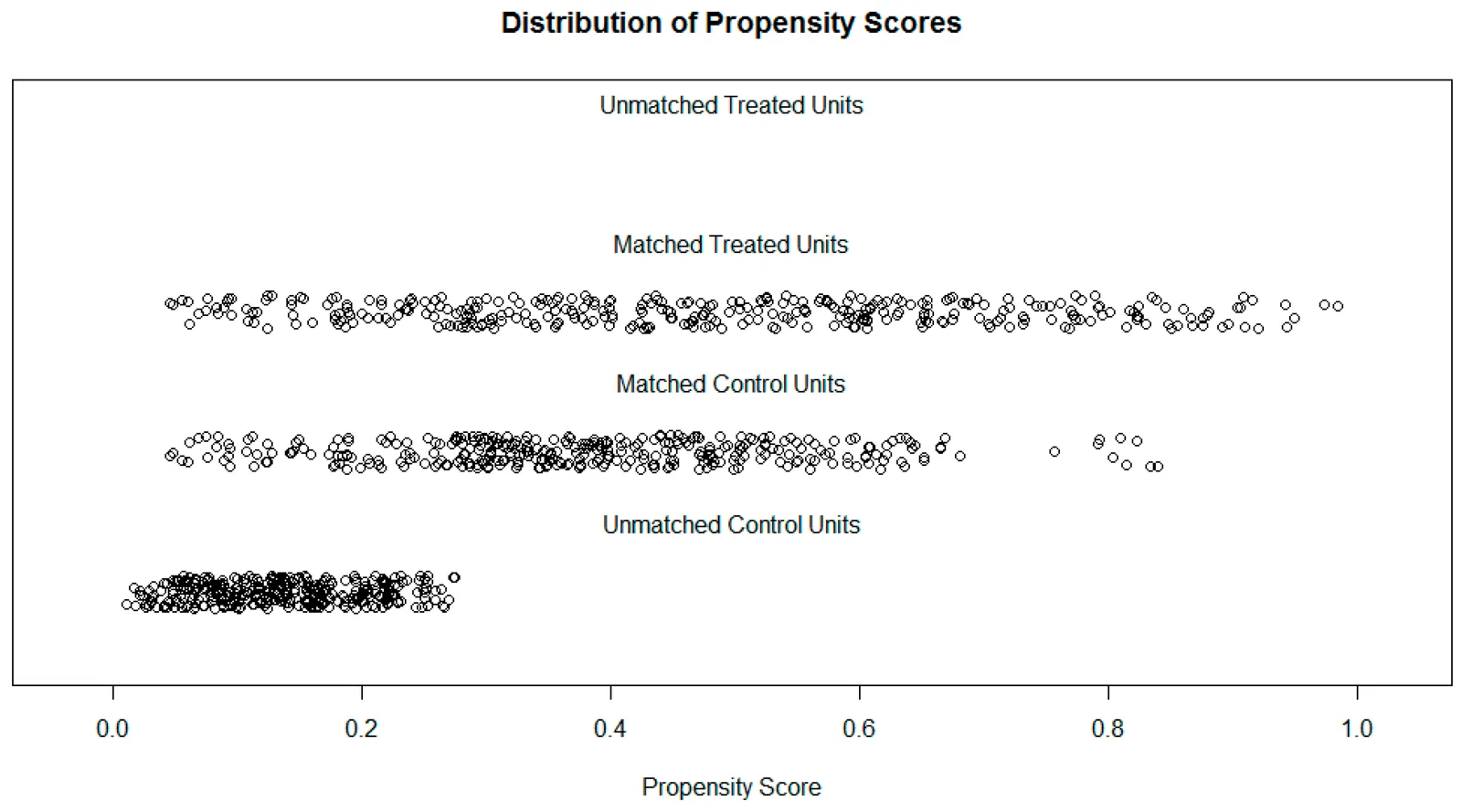
The distribution of propensity score before and after PSM analysis (std.mean diff 0.957–0.418).

Figure 1 illustrates the distribution of propensity scores in both groups before and after PSM. After 1:1 nearest-neighbor matching, the propensity score distributions of the matched groups showed improved overlap compared with the pre-matching distributions. This suggests that PSM improved baseline comparability between the two groups based on the measured covariates, although residual imbalance remained for some variables, particularly age and BMI.

**Figure 2:**
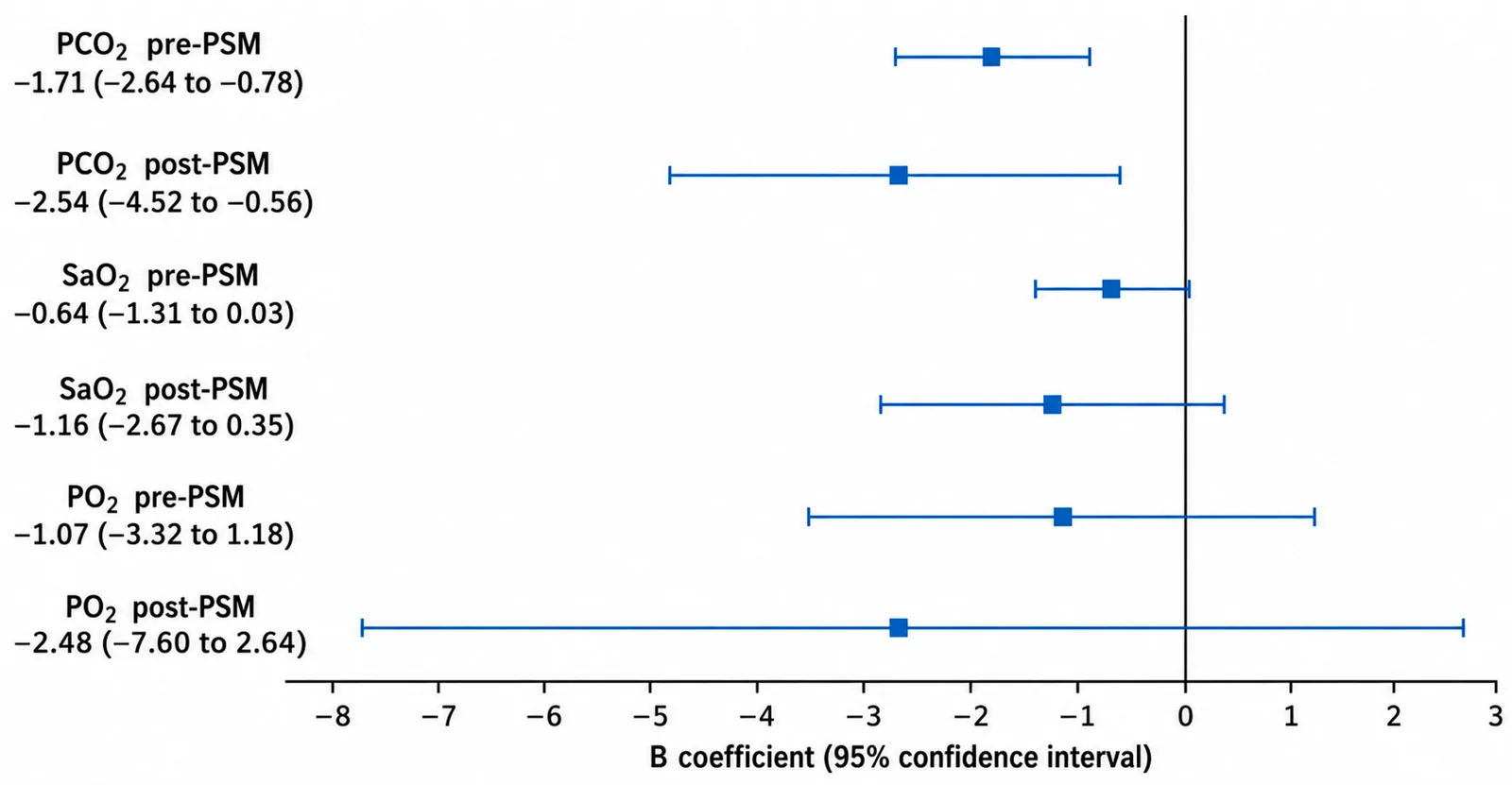
Association between coexisting hypertension and diabetes and blood gas analysis indicators before and after propensity score matching. (Note: Blue squares represent the estimated B coefficient, and horizontal blue lines represent the corresponding 95% confidence interval (CI)).

The association between the comorbidity status (COPD with hypertension and diabetes) and key blood gas parameters, before and after propensity score matching, is visually summarized in the provided Figure 2, which presents the B coefficients along with their 95% confidence intervals. For the parameter PCO_2_, the association before matching (pre-PSM) was a B coefficient of −1.71 (95% CI: −2.64 to −0.78). After matching (post-PSM), the point estimate indicated a stronger negative association, with a B coefficient of −2.54 (95% CI: −4.52 to −0.56). For the parameter SaO_2_, the association before matching was a B coefficient of −0.64 (95% CI: −1.31 to 0.03). After matching, the point estimate suggested a slightly stronger negative association (B = −1.16), but the confidence interval remained wide and crossed the null value (95% CI: −2.67 to 0.35), indicating statistical non-significance. For the parameter PO_2_, the association before matching was a B coefficient of −1.07 (95% CI: −3.32 to 1.18). After matching, the point estimate also suggested a negative association (B = −2.48), but with a wide confidence interval spanning from −7.6 to 2.64, which also includes the null value. Overall, Figure 2 indicates that coexisting hypertension and diabetes were consistently associated with lower PCO_2_ levels in elderly COPD patients, whereas no statistically significant associations were observed for SaO_2_ or PO_2_. The wider confidence intervals after matching, particularly for PO_2_, suggest greater uncertainty in the post-matching estimates. Therefore, these results should be interpreted cautiously as associations rather than causal effects.

Since residual imbalance in age and BMI persisted after propensity score matching, sensitivity analysis adjusting for age and BMI was conducted to evaluate the robustness of the findings. The sensitivity analysis yielded highly consistent effect estimates for all blood gas endpoints compared with the primary post-matching model (see Supplementary Table S2).

## 4. Discussion

In this retrospective cross-sectional study of elderly patients with COPD, we examined the association between coexisting hypertension and diabetes and arterial blood gas parameters using propensity score matching. The main finding was that COPD patients with both hypertension and diabetes showed lower arterial PCO_2_ levels than COPD-only patients after matching and adjustment for available covariates. In contrast, the associations with oxygen-related indicators, including PO_2_ and SaO_2_, were not statistically significant.

Previous studies examining cardiometabolic comorbidities and arterial blood gas abnormalities in COPD have produced evidence that is only partly comparable with our findings. Lin et al. [31] reported that patients with acute exacerbation of COPD and newly diagnosed type 2 diabetes had a lower PO_2_ level than patients without diabetes. This finding supports a relationship between diabetes and impaired pulmonary gas exchange, but their study did not demonstrate a significant independent reduction in PCO_2_. In addition, the relationship between hypertension and arterial blood gas parameters has also been insufficiently characterized. Arslan et al. [32] found that night-time systolic blood pressure was positively correlated with PCO_2_, PO_2_, pH, and HCO_3_^−^ in patients with COPD. However, patients with established hypertension were excluded from their study, and the analysis evaluated correlations between ambulatory blood pressure and blood gas parameters within a COPD population rather than directly comparing patients with and without hypertension. Therefore, the positive correlation between night-time blood pressure and PCO_2_ reported in that study is not necessarily inconsistent with the lower PCO_2_ observed in our patients with diagnosed hypertension and diabetes. In addition, Hu et al. [33] identified diabetes and PCO_2_ as separate predictors of adverse outcomes in acute exacerbations of COPD, but did not determine whether diabetes itself was associated with increased or decreased PCO_2_.

The existing clinical literature has proposed several hypothetical physiological pathways linking hypertension, diabetes and altered carbon dioxide levels, including diabetic autonomic neuropathy, systemic inflammatory activation and metabolic acid–base compensation [13,34,35]. However, these pathways cannot be verified in the present analysis. Our dataset lacks critical ventilatory and acid–base markers, such as respiratory rate, minute ventilation, pH, bicarbonate, lactate, inflammatory markers, or autonomic function. With only isolated PCO_2_ measurements available, we cannot draw definitive physiological interpretations for the observed intergroup difference in PCO_2_. All above hypotheses are only background summaries from prior studies, rather than validated explanations for our observational association.

The identification of a lower PCO_2_ in this complex patient population carries important clinical messages. From a clinical perspective, these results support the value of comprehensive arterial blood gas assessment in elderly COPD patients with multiple comorbidities. Pulse oximetry provides useful information on oxygen saturation, but it cannot directly assess carbon dioxide retention or reduction. A seemingly “normal” oxygen saturation might mask underlying ventilatory disturbances. Arterial Blood Gas (ABG) analysis [11,35,36], which provides direct PCO_2_ measurement, could be considered a valuable tool for a comprehensive assessment in these individuals. In patients with COPD and cardiometabolic comorbidities, PCO_2_ may provide additional information regarding ventilatory and metabolic status. Lower PCO_2_ may serve as a potential clinical signal of physiological complexity in COPD patients with coexisting hypertension and diabetes, although its relationship with autonomic dysfunction, systemic inflammation, or overall disease burden requires further investigation. It may identify a patient subgroup at higher risk for adverse outcomes, such as cardiac arrhythmias or increased mortality, warranting more vigilant management. This physiological insight could inform treatment strategies. For instance, a low level of PCO_2_ might necessitate caution with therapies that could further depress ventilation (e.g., high-concentration oxygen in certain contexts, sedatives) and highlights the importance of optimizing management of the underlying comorbidities themselves. However, the clinical implications of lower PCO_2_ in this population remain uncertain. It should not be assumed to represent a distinct ventilatory phenotype without supporting physiological measurements. Instead, it may serve as a signal that patients with COPD, hypertension, and diabetes have a more complex cardiopulmonary and metabolic profile that requires careful clinical evaluation.

The absence of statistically significant differences in PO_2_ and SaO_2_ should likewise not be interpreted as proof that coexisting hypertension and diabetes have no relevance to oxygenation. Both parameters are influenced by supplemental oxygen, acute exacerbation status, ventilation-perfusion mismatch, hemoglobin concentration, pulmonary vascular disease, and measurement timing. The relatively wide variability of PO_2_ and SaO_2_ in clinical populations may also reduce the precision of between-group estimates.

In the present analysis, PSM improved the balance of several measured sociodemographic and clinical variables between the two groups, including sex, marital status, and body temperature. The reduction in covariate-specific standardized mean differences and the improved overlap of propensity score distributions after matching suggest that the matched cohort had better baseline comparability than the original sample. This process may have reduced, to some extent, bias arising from observed confounding factors. However, it must be clearly stated that PSM failed to eliminate differences in all baseline characteristics. Key clinical variables, such as age and BMI, still showed statistically significant differences between groups after matching. This finding is crucial as it reveals the inherent limitations of the PSM method: its efficacy strictly depends on the observed covariates and the overall structure of the study sample. PSM can only balance variables that have been measured and correctly included in the propensity score model; it is ineffective against unmeasured variables or model misspecification. The residual confounding observed in this study may stem from several aspects. Firstly, sample size limitations could be a factor. Although the initial sample size was substantial, during 1:1 nearest-neighbor matching, there might have been insufficient overlap in the extreme values of age and BMI between the two groups, preventing ideal matches for some individuals and thus preserving these differences. Secondly, and most notably, is the possibility of unmeasured confounding factors. Observational databases may not capture all variables related to both patient grouping and outcomes. For instance, disease severity (e.g., COPD GOLD grade), lung function, duration of illness, medication adherence, lifestyle factors (e.g., pack-years of smoking, diet, physical activity level), and socioeconomic status are factors highly likely to influence both the risk of developing hypertension/diabetes comorbidities and the results of blood gas analysis [35]. Since these variables were not included in the initial propensity score model, PSM could not balance them, allowing them to persist as potential sources of residual confounding.

The contribution of this study lies in addressing a narrowly defined and understudied question: the association between the combined presence of hypertension and diabetes and arterial blood gas parameters in elderly COPD patients. Unlike previous studies that primarily examined comorbidity prevalence, spirometric impairment, or clinical outcomes, this analysis focused on PCO_2_, PO_2_, and SaO_2_ and attempted to reduce measured baseline differences using PSM. While our findings are informative, several limitations must be acknowledged. Firstly, the study compared COPD patients with both hypertension and diabetes with COPD-only patients. This design does not allow us to determine whether the observed association was driven primarily by hypertension, diabetes, or their combined presence. Future studies should include separate COPD plus hypertension and COPD plus diabetes groups, or formally test the interaction between hypertension and diabetes if the data permit. Secondly, the retrospective, cross-sectional nature of our analysis allows us to identify associations but does not establish causality. The directionality and long-term dynamics of the observed relationship require further investigation through longitudinal studies. Thirdly, after propensity score matching, the overall standardized mean difference decreased substantially from 0.957 to 0.418, yet residual imbalance in age and BMI could not be fully eliminated, which may introduce residual confounding and limit the ability to completely rule out the influence of these two covariates. Fourthly, our study was conducted using data from a single source. The patient characteristics and clinical practices might differ in other populations, which could affect the generalizability of our results. Therefore, future prospective studies incorporating more granular physiological measurements and longer follow-up are needed to confirm these findings and elucidate the causal pathways. Finally, we adopted ordinary linear regression after 1:1 propensity score matching. Theoretically, this approach does not explicitly model the matched pair structure. However, the matched pairs were constructed for covariate balance rather than repeated measurements of the same subject. Sensitivity analysis adjusted for age and BMI yielded consistent results, supporting the robustness of our findings. Future studies may adopt mixed-effects models to further verify relevant associations.

## 5. Conclusions

In this retrospective cross-sectional study of elderly patients with COPD, coexisting hypertension and diabetes were associated with lower arterial PCO_2_ levels after propensity score matching and adjustment for all available recorded covariates. In contrast, no statistically significant associations were observed for PO_2_ or SaO_2_. While these observational results indicate a statistical correlation between combined hypertension–diabetes comorbidity and altered PCO_2_, substantial unmeasured confounding persists due to the complete absence of critical COPD-related confounders, including FEV_1_, GOLD disease grading, smoking history, acute exacerbation status, long-term oxygen therapy, and regular medication records. Therefore, these findings must only be interpreted as tentative statistical associations rather than validated causal relationships. Future prospective longitudinal studies that systematically collect comprehensive pulmonary function, disease severity, treatment, and lifestyle covariates are urgently required to replicate these observations, quantify residual confounding, and further investigate potential physiological pathways linking cardiometabolic comorbidities to ventilatory gas exchange.

## Figures and Tables

**Table 1:** Basic socio-demographic variables (*n* = 1030).

Characteristic	Number	Percent (%)
Age (years)	77.54 ± 9.36	
60–64	74	7.2
65–69	157	15.2
70–74	202	19.6
75-	597	58.0
Sex		
Women	198	19.2
Men	832	80.8
Marital status		
Married	804	78.1
Widowed/Others	226	21.9
BMI (kg/m^2^)	21.31 ± 3.24	
Underweight (<18.5)	174	16.8
Normal weight (18.5–24.9)	738	72.1
Overweight (25.0–29.9)	100	9.6
Obesity (>30)	18	1.5
Diabetic with coexisting hypertension and diabetes		
Yes	340	33.3
None	690	66.7
Temperature (°C)	36.60 ± 0.55	
Pulse (beats per minute)	87.02 ± 18.09	
PCO_2_ (mmHg)	45.26 ± 14.37	
SaO_2_ (%)	92.79 ± 9.85	
PO_2_ (mmHg)	84.41 ± 32.86	

**Table 2:** Baseline characteristics and covariate balance before and after propensity score matching.

Variables	Before PSM Analysis (*n* = 1030)		χ^2^	*p*	After PSM Analysis (*n* = 680)		χ^2^	*p*
	Comorbidity Group (*n* = 340)	COPD-Only Group (*n* = 690)			Comorbidity Group (*n* = 340)	COPD-Only Group (*n* = 340)		
Sex			0.90	0.343			0.145	0.703
Women	71 (35.9)	127 (64.1)			71 (51.4)	67 (48.6)		
Men	269 (32.3)	563 (67.7)			269 (49.6)	273 (50.4)		
Marital status			7.76	0.005			2.889	0.089
Married	248 (30.8)	556 (69.2)			248 (48.2)	267 (51.8)		
Widowed/Others	92 (40.7)	134 (59.3)			92 (55.8)	73 (44.2)		
	Mean ± SD	Mean ± SD	t	*p*	Mean ± SD	Mean ± SD	t	*p*
Age	81.87 ± 9.86	75.41 ± 8.31	121.60	<0.001	81.87 ± 9.86	79.57 ± 8.25	10.91	0.001
BMI	22.79 ± 3.55	20.58 ± 2.80	117.89	<0.001	22.79 ± 3.55	21.91 ± 2.55	13.80	<0.001
Temperature	36.59 ± 0.54	36.60 ± 0.55	0.226	0.634	36.59 ± 0.54	36.59 ± 0.53	0.0002	0.991
Pulse	85.06 ± 17.53	87.98 ± 12.30	5.954	0.015	85.06 ± 17.53	86.83 ± 17.85	1.698	0.193

Notes: The comorbidity group refers to “COPD patients with coexisting hypertension and diabetes”, whereas the COPD-only group refers to “COPD-only patients” without hypertension or diabetes. SMD for each covariate before and after matching is shown in Supplementary Table S1. Values are presented as mean ± standard deviation or *n* (%).; PSM, propensity score matching; COPD, chronic obstructive pulmonary disease.

## Data Availability

The data presented in this study are not publicly available due to privacy and ethical restrictions. De-identified data may be available from the corresponding author upon reasonable request and with approval from the relevant ethics committee or institution.

## Supplementary Materials

The following supporting information can be downloaded at: https://www.mdpi.com/article/10.3390/healthcare14162500/s1, Table S1: The SMD for each covariate before and after matching; Table S2: Association between coexisting hypertension and diabetes and blood gas analysis indicators after propensity score matching and adjustment for age and BMI.
