# Supplementary material for: Association of Coexisting Hypertension and Diabetes with Blood Gas Analysis Indicators in COPD Patients: A Propensity Score-Matched Analysis
Source: Healthcare (Basel). 2026 Aug 12;14(16):2500. doi: 10.3390/healthcare14162500 (PMC13512700; doi:10.3390/healthcare14162500)
Supplement: Supplementary file 1 [file healthcare-14-02500-s001.zip › healthcare-4402217-supplementary.pdf]

Table S1: The SMD for each covariate before and after matching.

Std. Mean	pre	post
sex	0.0609	0.0289
marital status	0.1719	0.1258
age	0.6559	0.2335
bmi	0.6211	0.248
temperature	0.0319	0.0009
pulse	0.1665	0.1008

Note: All data are expressed as absolute values.

Table S2: Association between coexisting hypertension and diabetes and blood gas analysis indicators after propensity score matching and adjustment for age and BMI.

Outcome	B	95% CI
PCO_2	-2.436	-4.614, -0.258
SaO_2	-1.002	-2.521, 0.517
PO_2	-1.525	-6.670, 3.620
